# A Worm's Tale or Why to Avoid the Raccoon Latrine: A Case of *Baylisascaris procyonis* Meningoencephalitis

**DOI:** 10.1155/2022/5199863

**Published:** 2022-08-21

**Authors:** Adam E. Goldman-Yassen, Anna Derman, Rebecca Pellett Madan, Alireza Radmanesh

**Affiliations:** ^1^Department of Radiology, Children's Healthcare of Atlanta, 1405 Clifton Rd. NE Atlanta, GA 30322, USA; ^2^Department of Radiology and Imaging Sciences, Emory University School of Medicine, 201 Dowman Dr, Atlanta, GA 30322, USA; ^3^Department of Radiology, Maimonides Medical Center, 4802 Tenth Avenue 3rd floor, Brooklyn, NY 11219, USA; ^4^Department of Pediatrics, NYU Langone Medical Center, 550 First Avenue New York, NY 10016, USA; ^5^Department of Radiology, NYU Langone Medical Center, 550 First Avenue, New York, NY 10016, USA

## Abstract

The raccoon roundworm *Baylisascaris procyonis* (*B. procyonis*) may infect humans to cause severe or fatal meningoencephalitis, as well as ocular and visceral larva migrans. Young children are at greater risk for cerebral larva migrans with severe meningoencephalitis, and early empiric therapy may improve outcomes. Familiarity with characteristic brain imaging findings may prompt earlier diagnosis, particularly in the setting of CSF eosinophilia. We report a case of a 19-month-old boy who presented with truncal ataxia and was found to have peripheral and CSF eosinophilia. MRI demonstrated symmetric, confluent T2 hyperintense signal in the cerebral and cerebellar deep white mater, which helped differentiate *B. procyonis* meningoencephalitis from other infectious and non-infectious causes of eosinophilic meningoencephalitis. Early recognition and treatment of *B. procyonis* meningoencephalitis are important for improved outcomes, and careful review of neuroimaging can play a critical role in suggesting the diagnosis.

## 1. Case Report

A 19-month-old unimmunized boy presented to the hospital with a five-day history of truncal ataxia. The child was previously healthy and had met all developmental milestones at appropriate intervals. He began to walk independently at 12 months of age and was running by 15 months of age. Three weeks prior to presentation, his mother noted the onset of irritability and disrupted nighttime sleep. Five days prior to presentation, the child developed an axillary temperature of 38° C and inability to sit or walk unassisted. His mother also noted a sudden deceleration in language acquisition. The child's symptoms were initially attributed to bilateral otitis media, but symptoms failed to improve with antibiotics. He was subsequently brought to the pediatric emergency department for evaluation.

In the emergency department, the child was found to have a temperature of 36.7°C, heart rate of 116 beats/min, respiratory rate of 29 breaths/min, and blood pressure of 101/68 mmHg. Room air oxygen saturation was 98%. The child was awake, alert, and verbal. The child had normal muscle bulk and tone, good antigravity strength in all extremities, and intact deep tendon reflexes in the upper and lower extremities. Neurological exam was significant for profound truncal ataxia. The child could not walk or sit unassisted. He showed a preference for use of his left hand but had no dysmetria when reaching for objects. The remainder of his exam was unremarkable.

Laboratory analysis on hospital day 0 was significant for an elevated peripheral white blood cell count (WBC) of 24,000 cells/*μ*L, with 18% eosinophils (normal < 6%). Erythrocyte sedimentation rate was also elevated to 39 mm/hr. Cerebrospinal fluid on hospital day 0 showed WBC count of 13 cells/mm^3^ (normal < 5 cells) with 29% eosinophils, red blood cell (RBC) count 2 of cells/mm^3^, glucose of 50 mg/100 mL (normal 50-80), and protein of 33 mg/dL (normal 15-60). CSF was again sampled on hospital day 4 and showed WBC count of 31 cells/mm^3^ (41% eosinophils), RBC count of 0 cells/mm^3^, glucose 48 mg/100 mL, and protein 33 mg/dL.

**Figure 1:**
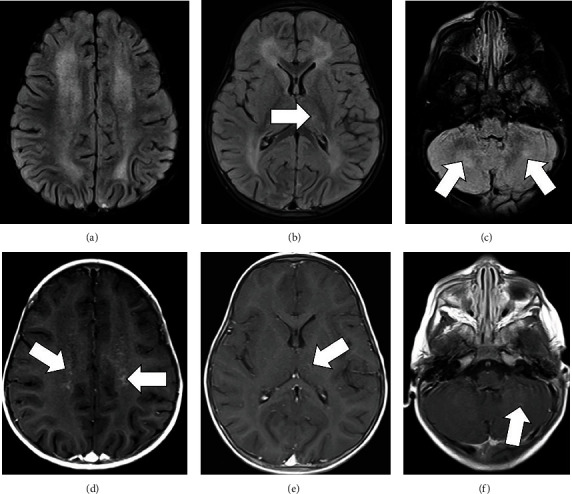
MRI obtained on admission demonstrating diffuse confluent symmetric T2 FLAIR hyperintensities in the supratentorial periventricular and deep white matter, sparing the subcortical U fibers, and in bilateral cerebellar hemispheres around the deep nuclei (a–c). A focus of T2 FLAIR hyperintensity is noted in the lateral left thalamus (arrow in (b)). Postcontrast images reveals punctiform and curvilinear enhancement centered in bilateral supratentorial periventricular white matter with a perivascular distribution pattern (arrows in (d)). Foci of faint and curvilinear enhancement (arrow in (f)) were also present in bilateral cerebellar white matter. Small curvilinear enhancement was noted in the lateral left thalamus, likely reflective of a vein or a perivenous process (arrow in (e)).

Brain MRI demonstrated diffuse confluent, symmetric T2 FLAIR hyperintensity in the supratentorial periventricular and deep white matter and bilateral cerebellum around the deep cerebellar nuclei (Figures 1(a)–1(c)). Postcontrast images revealed punctiform and curvilinear enhancement, mainly in bilateral cerebral and cerebellar periventricular white matter with a pattern suggestive of perivascular distribution (Figures 1(d)–1(f)). Spinal MRI was normal (not shown).

Extensive testing of CSF for viral, bacterial, fungal, amoebic, and mycobacterial pathogens was negative, and the predominance of eosinophils in both peripheral blood and CSF raised concern for eosinophilic meningoencephalitis secondary to parasitic infection. After consultation with parasitology experts from the Centers for Disease Control and Prevention (CDC) and from the New York City Department of Health and Mental Hygiene (NYC DOHMH), the patient began empiric treatment for meningoencephalitis secondary to cerebral larva migrans with oral albendazole (40 mg/kg/day) and intravenous methylprednisolone (5 mg/kg/day).

**Figure 2:**
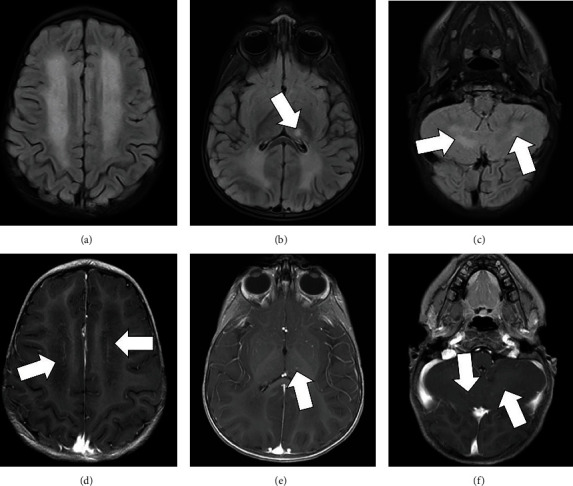
MRI obtained 10 days after admission demonstrating mild progression in diffuse white matter T2 FLAIR hyperintensity (a–c) with slightly increased involvement of the left lateral thalamus (arrow in (b)). Previously noted punctiform enhancement in the supratentorial periventricular white matter has nearly resolved with minimal residual curvilinear enhancement, possibly vessels (arrows in (d)). Small curvilinear enhancement is again seen in the left thalamus (arrow in (e)). Prior curvilinear enhancement in the cerebellum had resolved with interval development of few new punctiform cerebellar enhancing foci (arrows in (f)).

Follow-up imaging obtained 10 days after admission demonstrated mild progression in diffuse white matter T2 FLAIR hyperintense inflammatory process (Figures 2(a)–2(c)) with slightly increased involvement in the left lateral thalamus (Figure 2(b)). Previously noted punctiform enhancement in the supratentorial periventricular white matter had nearly resolved with minimal residual curvilinear enhancement (Figure 2(d)). Prior curvilinear enhancement in the cerebellum had also resolved with interval development of few new punctiform cerebellar enhancing foci (Figure 2(f)).

By hospital day 11, the child showed marked improvement and was discharged to home to continue oral albendazole and prednisone. Neurological exam at the time of hospital discharge was significant only for wide-based gait, but the child was otherwise able to ambulate and sit independently and was felt by his parents to have returned to his normal level of activity and verbal communication. The child was seen for outpatient follow up approximately one month after initial onset of symptoms and continued to show a subtle, wide-based gait. Repeat CBC at this time showed complete resolution of peripheral leukocytosis and eosinophilia. The child received a total of two months of oral albendazole therapy and corticosteroids. Although a follow-up MRI had been planned for three months after initial presentation, the child did not return to clinic, and this imaging was not completed.

CSF and serum from the patient were sent to the Division of Parasitic Diseases and Malaria (DPDM) at the CDC for *Baylisascaris procyonis* antibody testing by Western blot [1]. This assay detects *Baylisascaris*-specific antibodies to a recombinant protein (rRAG1) derived from *B. procyonis* and, of note, does not cross react with *Toxocara*-specific antibodies [2]. Testing revealed a positive *B. procyonis* Western blot result from serum but negative Western blot result from CSF. We did not have access to reported Western blot band intensity, nor did we have access to convalescent serum or additional CSF for repeat testing. Testing for *Toxocara* by the CDC was negative. Based on the child's presentation, the remaining negative diagnostic tests, characteristic imaging, and positive serum Western blot result, the child was presumed to have meningoencephalitis secondary to *B. procyonis*. Although the two older children in the household were not available for serological testing, they also received preemptive albendazole therapy.

The child described in this case lived in Brooklyn, New York, with his parents and two older siblings. The child was reported to play outdoors frequently in the alley next to his home and also spent time in upstate New York. Although a comprehensive environmental evaluation by the New York City Department of Health and Mental Hygiene (NYC DOHMH) did not identify a raccoon latrine in close proximity to the child's environment, raccoons are ubiquitous throughout urban environments. *B. procyonis* eggs are frequently shed by animals other than raccoons, including domesticated dogs, and are extremely resistant to environmental decontamination efforts [3].

## 2. Discussion

*B. procyonis* is commonly found in the small intestines of raccoons throughout the United States, including raccoons in New York City [4–9]. It can cause severe or fatal encephalitis (neural larval migrans) in a variety of birds and mammals, including human, as well as human ocular and visceral larval migrans [4, 7, 10–13]. Humans become infected by ingesting materials contaminated with raccoon feces containing *B. procyonis* eggs, most often soil [14]. Young children are particularly at risk for infection due to behaviors such as pica and geophagia and routinely placing potentially infected fingers and other objects into their mouths [7, 12, 15]. Once consumed, the larvae migrate to various tissues, including viscera, the eyes, and the central nervous system from the gastrointestinal tract [16]. The severity of neurological disease in humans depends on the number of eggs ingested and the number of larvae that migrate to the CNS. Larvae in the CNS cause an inflammatory reaction along with tissue injury and can become encapsulated and form granulomas [11]. In addition to direct injury by migrating larvae, toxic eosinophil degranulation releases harmful proteins into the CNS, including eosinophil-derived neurotoxin [17].

*B. procyonis* meningoencephalitis demonstrates remarkably similar imaging features across various case reports. Characteristic findings on MRI include diffuse symmetric deep white matter injury in the form of T2/FLAIR hyperintensities in the supratentorial periventricular and deep white matter and in bilateral cerebellar hemispheres around the deep cerebellar nuclei [15, 17–20]. There is usually sparing of the subcortical U fibers and superimposed relatively symmetric punctiform and hazy ill-defined enhancement, suggesting a perivascular process [6, 20]. Neurotoxins resulted from eosinophil degranulation can enter the CNS and contribute to diffuse white matter injury with Baylisascaris infection [17]. Interestingly, similar imaging features have been described with idiopathic hypereosinophilic syndrome manifesting with CNS vasculitis, possibly related to the same mechanism of tissue injury [21].

Although the MRI appearance of *B. procyonis* encephalitis is not specific, the presence of peripheral and CSF eosinophilia in combination with the above described typical white matter changes on brain MRI or signs of a neuroretinitis strongly suggests *B. procyonis* infection. The differential diagnosis for CSF eosinophilia (defined as >10 eosinophils/mm^3^ in the CSF and/or eosinophils > 10% of cell count) includes infectious, toxic, inflammatory, and neoplastic etiologies. Unlike *Baylisascaris*, other parasitic infections tend to present with more focal and asymmetric abnormalities on brain MRI without symmetric deep white matter involvement as is common with *B. procyonis*. *Angiostrongylus cantonensis* is the most common cause of eosinophilic meningitis worldwide, endemic to Southeast Asia, is associated with rat infection, and can demonstrate multiple prominent Virchow-Robin spaces, subcortical enhancing lesions, and T2 hyperintense lesions in the periventricular parenchyma of the brain, as well as linear enhancement in the leptomeninges [22, 23]. *Gnathostoma spinigerum*, an intestinal parasite of cats and dogs in Southeast Asia, Europe, South America, Africa, and the Middle East, can demonstrate foci of parenchymal hemorrhage (including hemorrhagic tracts) and multiple enhancing nodules in the brain, as well as myelitis [24]. *Toxocara canis* larvae invade the CNS of animals and are rarely associated with clinical CNS disease in humans, revealing enhancing subcortical and white matter lesions with imaging features sometimes similar to *Baylisascaris* [25]. Neurocysticercosis involvement of CNS includes acute encephalitis with multifocal brain lesions and basilar meningitis [26]. *Coccidioides immitis*, a fungus, is the most common cause of eosinophilic meningitis in the United States and can demonstrate leptomeningeal enhancement, characteristically localized in the basal cisterns with associated subarachnoid bleeding [27, 28]. Other entities that may cause CSF eosinophilia such as neoplasm (Hodgkin's disease and leukemia), drug effects, and reaction to foreign bodies are not commonly associated with encephalitis or white matter disease [29–31].

Importantly, imaging findings and demographics of *B. procyonis* meningoencephalitis may be identical to certain inborn errors of metabolism, including metachromatic leukodystrophy (MLD), a lysosomal storage disease typically affecting patients in the first three years of life [32]. Globoid Cell Leukodystrophy (or Krabbe's disease) may share the same imaging features but the early infantile form usually presents before 6 months of age, rapidly progressing to death within 1 year [33]. Both MLD and Krabbe's disease may present with diffuse symmetric confluent T2 hyperintense areas of demyelination involving the deep supratentorial white matter and cerebellum. These are usually nonenhancing but may rarely enhance in MLD [32, 34]. Disorders such as organic acidemias may first become clinically evident during an unrelated acute infection, such as a cold or flu, potentially confusing the clinical picture. Urea cycle disorders, nonketotic hyperglycinemia, peroxisomal disorders, maple syrup urine disease, and the effects of previous radiation or chemotherapy could also mimic imaging findings of *B. procyonis* encephalitis [15]. However, unlike *Baylisascaris* infection, none of these conditions routinely present with peripheral and CSF eosinophilia.

Imaging features in our case mitigated against alternative diagnosis such as leukemia, Hodgkin lymphoma, or fungal infection with *Coccidioides immitis,* as these entities do not have a specific predilection for the deep white matter. Additionally, pattern of white matter injury in our case was inconsistent with acute disseminated encephalomyelitis (ADEM), which is often a multifocal and asymmetric process involving both grey and white mater, including the deep grey nuclei [35].

A diagnosis of *B. procyonis* encephalitis should be considered in the setting of eosinophilic encephalitis and a history of potential exposure to raccoon feces, especially in children. Diagnostic findings include CSF eosinophilic pleocytosis, peripheral eosinophilia, symmetric confluent deep white matter abnormalities on MRI, and positive serologic testing of CSF and serum [10]. Western blot testing of CSF and serum using recombinant *B. procyonis* antigen remains the preferred method of testing and is available through the United States CDC DPDM and the National Reference Center for Parasitology in Montreal, Canada [2]. Brain parenchymal injury progresses rapidly if the *B. procyonis* encephalitis is left untreated; hence, anthelmintic medication should be administered as soon as the infection is suspected to prevent catastrophic sequalae [36]. Treatment of symptomatic patients with anthelmintic or anti-inflammatory drugs often will not improve outcomes because CNS damage can occur before patients become symptomatic [37, 38]. Urgent anthelmintic treatment started just after a possible infection may prevent clinical disease by killing larvae before they enter the CNS.

In conclusion, *B. procyonis* meningoencephalitis should be considered in patients with symmetric diffuse supratentorial and cerebellar deep white matter injury in the setting of peripheral and CNS eosinophilia. Early recognition and correct treatment of this disease may prevent catastrophic CNS sequelae.

## Data Availability

The patient data/case information supporting this case report is not available due to patient privacy. The other information cited in the study is from previously reported studies cited in the references.
